# Role of Molecular Charge in Nucleocytoplasmic Transport

**DOI:** 10.1371/journal.pone.0088792

**Published:** 2014-02-18

**Authors:** Alexander Goryaynov, Weidong Yang

**Affiliations:** Department of Biology, Temple University, Philadelphia, Pennsylvania, United States of America; Consejo Superior de Investigaciones Cientificas, Spain

## Abstract

Transport of genetic materials and proteins between the nucleus and cytoplasm of eukaryotic cells is mediated by nuclear pore complexes (NPCs). A selective barrier formed by phenylalanine-glycine (FG) nucleoporins (Nups) with net positive charges in the NPC allows for passive diffusion of signal-independent small molecules and transport-receptor facilitated translocation of signal-dependent cargo molecules. Recently, negative surface charge was postulated to be another essential criterion for selective passage through the NPC. However, the charge-driven mechanism in determining the transport kinetics and spatial transport route for either passive diffusion or facilitated translocation remains obscure. Here we employed high-speed single-molecule fluorescence microscopy with an unprecedented spatiotemporal resolution of 9 nm and 400 µs to uncover these mechanistic fundamentals for nuclear transport of charged substrates through native NPCs. We found that electrostatic interaction between negative surface charges on transiting molecules and the positively charged FG Nups, although enhancing their probability of binding to the NPC, never plays a dominant role in determining their nuclear transport mode or spatial transport route. A 3D reconstruction of transport routes revealed that small signal-dependent endogenous cargo protein constructs with high positive surface charges that are destined to the nucleus, rather than repelled from the NPC as suggested in previous models, passively diffused through an axial central channel of the NPC in the absence of transport receptors. Finally, we postulated a comprehensive map of interactions between transiting molecules and FG Nups during nucleocytoplasmic transport by combining the effects of molecular size, signal and surface charge.

## Introduction

In eukaryotic cells, transport of macromolecules between the nucleus and cytoplasm is mediated by the nuclear pore complexes (NPCs) embedded in the double-membrane nuclear envelope (NE) [1]–[5]. The NPC is composed of approximately thirty different nucleoporins (Nups), each of which is present in approximately eight copies. Approximately one third of these Nups are intrinsically disordered proteins with multiple phenylalanine-glycine (FG) repeats that form a highly selective barrier due to weak hydrophobic interactions between them [6]–[8]. Currently, it is well-recognized that molecular size and the specific signal carried by the molecules act as the primary criteria that determine the transport mode for transiting molecules through the selective barrier in the NPC [9]–[13]. In particular, molecules which are smaller than 40 kDa and lack specific localization signals (either a lysine-rich nuclear localization sequence (NLS) for nuclear import or a leucine-rich nuclear export signal (NES) for nuclear export) passively diffuse through the NPC without consuming chemical energy. In contrast, cargo molecules that carry an NLS or an NES are capable of forming complexes with transport receptors to conduct facilitated diffusion through the NPC via hydrophobic interactions between transport receptors and the FG Nups, after which chemical energy is usually required for dissociation of transiting cargo complexes [14]–[16].

In addition to the above criteria of molecular size and specific signal, negative surface charge was recently suggested to act as another selection criterion for nucleocytoplasmic transport [17]. In particular, the unfolded FG Nups possess net positive charges and thus create a positively charged selective barrier in the NPC, which could repel (or attract) positively (or negatively) charged incoming cargo molecules because of the simple rule of electrostatic force: repulsion between like charged particles and attraction between opposite charged particles. This proposed charge-driven selection mechanism is rooted in an assumption that the FG Nups interact with each other to form a homogenous mesh and fully fill the NPC, as suggested in the selective phase/hydrogel meshwork model [18]–[19]. Additionally, in this model, both passive and facilitated transports are postulated to share spatial pathways through this meshwork.

However, several other models have proposed that passive and facilitated transport have distinct spatial transport paths, with small molecules passively diffusing through a central channel in the NPC [20]–[21]. Recently, experimental measurements demonstrated that passive diffusion and facilitated transport indeed follow distinct transport routes through the NPC and suggested that the filaments of FG Nups may not form a homogenous meshwork-like barrier in the NPC [22]–[30]. Of particular note, novel three-dimensional (3D) mappings of the transport pathways of simultaneous passive and facilitated transport in native NPCs clearly revealed that a single central channel serves as the major diffusion conduit for passive diffusion of signal-independent small molecules; meanwhile, receptor-facilitated translocation preferentially goes through the periphery around this central channel [27]–[31]. A recent theoretical estimate of the spatial distribution of charges in FG-Nup-formed barriers also suggested two distinct charged transport zones in the NPC [32]. Furthermore, our recent data revealed that this heterogeneous spatial distribution of FG filaments in the NPC can be regulated by transport receptors and energy indicators [27]. Therefore, the inhomogeneous and changeable configuration of the charged FG barrier could provide more complex charged environments than previously thought for passive and facilitated transport in the NPC.

**Figure 1:**
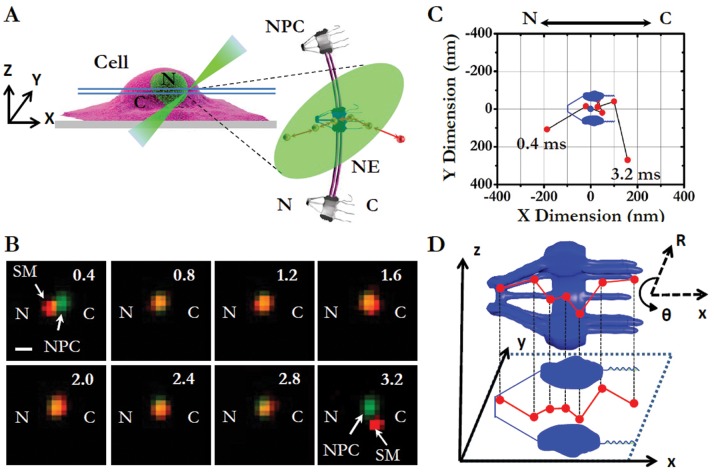
Transport of single molecules through a single NPC tracked by SPEED microscopy and further characterized with 2D to 3D deconvolution algorithms. (A) Diagram of the illumination of a single GFP-NPC (blue) and the tracking of single fluorescent in-transit molecules (red dots) through this NPC in cells by SPEED microscopy. SPEED microscopy generates an inclined illumination point spread function (iPSF forms an angle of 45° to the z direction) in the focal plane (between the double light blue lines) at the equatorial plane of a cell nucleus. C, cytoplasm. N, nucleus. (B) A well-isolated fluorescent spot of a single GFP-NPC (green) overlaid with a single in-transit fluorescent molecule (SM, red spot, here using Alexa Fluor 647 labeled single GFP molecule as an example) captured by SPEED microscopy. A typical nuclear transport event of a single molecule (red spot) from the nucleus to the cytoplasm is captured at 0.4 ms per frame. Numbers denote time in milliseconds. Scale bar, 1 µm. (C) Using a 2D Gaussian fit of isolated fluorescent spots, the trajectories of an in-transit molecule (red dots) and the centroid of the NPC (the blue dot) were obtained and then overlaid onto the NPC architecture (blue). Clearly, the transiting molecule interacted with the NPC from 0.8 ms to 2.8 ms. N, the nucleoplasmic side of the NPC. C, the cytoplasmic side of the NPC. (D) The diagram illustrates the 2D to 3D deconvolution process. In principle, 2D spatial locations (x, y) projected from 3D locations (x, y, z) in a Cartesian coordinate system retain all of the information about the 3D locations that can also be encoded in a cylindrical coordinate system (R, θ, x). Following an established approach, here a deconvolution process (or a back-projection process) was set up between a simplified cylindrical coordinate system (R, constant, x) and the obtained 2D spatial locations (x, y). Detailed deconvolution process can be found in previous publications [27], [30].

**Figure 2:**
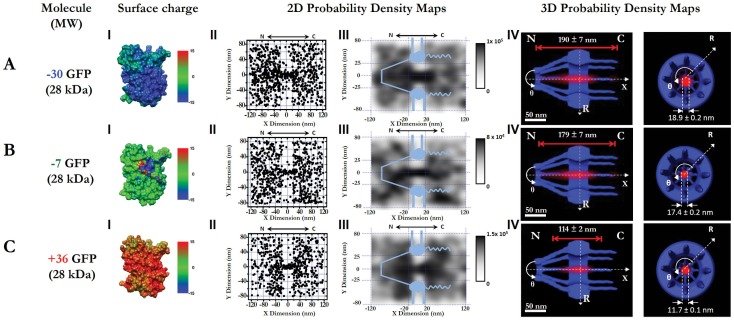
3D passive diffusion routes of differently charged GFPs. (A) −30 GFP. Calculated electrostatic surface potentials of GFP variants, ranging from −15 kT/e (dark blue) to +15 kT/e (dark red); neutral charge 0 is shown in green (I). Superimposed plots of thousands of localizations of single molecules located primarily within a rectangular area of 240×160 nm (II). N, the nucleoplasmic side of the NPC. C, the cytoplasmic side of the NPC. The locations in each 10×10 nm area were quantized and filtered with a Gaussian blur function to generate the 2D probability density map overlaid onto the NPC architecture (light blue). The highest density was 1×10^5^ locations/µm^2^, and the lowest was 0 locations/µm^2^, shown in gray (III). A 3D probability density map generated using 2D to 3D deconvolution algorithms (red cloud; brighter color indicates higher density) is shown in both side-view and a top-view orientations superimposed on the NPC architecture (blue). The length of the path and the diameter at the central plane of the NPC were measured and marked in nanometers (IV). N, the nucleoplasmic side of the NPC. C, the cytoplasmic side of the NPC. (B–C) Nucleocytoplasmic transport pathways of −7 GFP and +36 GFP.

**Figure 3:**
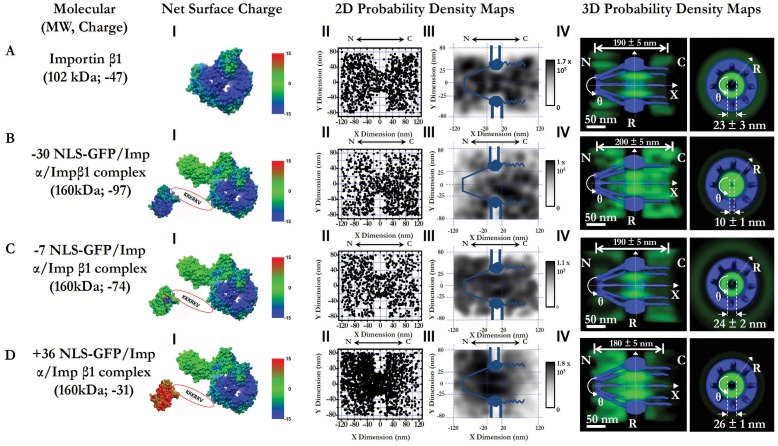
3D pathways of Imp β1 alone and Imp α/Imp β1 in complex with differently charged GFP cargo (−30 NLS-GFP), −7 NLS-GFP and +36 NLS-GFP). (A) Imp β1 alone. Calculated electrostatic surface potentials of Imp β1 range from −15 kT/e (dark blue) to +15 kT/e (dark red), and neutral charge 0 is shown in green (I). Superimposed plots of localizations of single molecules located primarily within a rectangular area of 240×160 nm (II). N, the nucleoplasmic side of the NPC; C, the cytoplasmic side of the NPC. The locations in each 10×10 nm area were quantized and filtered with a Gaussian blur function to generate the 2D probability density map overlaid onto the NPC architecture (light blue). The highest density was 1.7×10^5^ locations/µm^2^ and the lowest was 0 locations/µm^2^, shown in gray (III). A 3D probability density map (green cloud; brighter color indicates higher density) is shown in both side-view and a top-view orientations superimposed on the NPC architecture (blue). The length of pathway and the diameter at the central plane of NPC was measured and is labeled in nanometers. N, the nucleoplasmic side of the NPC. C, the cytoplasmic side of the NPC. (B–D) Nucleocytoplasmic transport pathways of −30NLS-GFP/Impα/Impβ1, −7NLS-GFP/Impα/Impβ1 and +36NLS-GFP/Impα/Impβ1.

**Figure 4:**
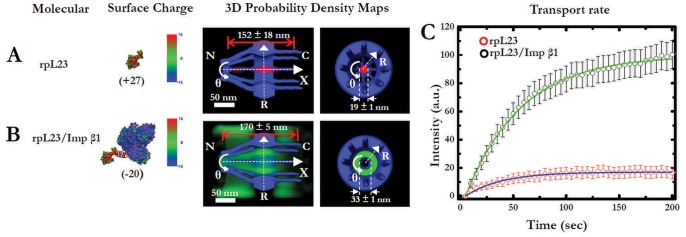
3D transport pathways of a positively charged ribosomal protein rpL23 through the NPC with and without Imp β1. (A) 3D transport routes of rpL23 alone. N, the nucleoplasmic side of the NPC. C, the cytoplasmic side of the NPC. (B) 3D transport routes of the cargo complex of rpL23/Imp β1. (C) Time-courses of net nuclear accumulations of rpL23 alone and rpL23/Imp β1. The graphs depict average intensity increase in the nuclei as the permeabilized cells were incubated with rpL23 (red open dots) or rpL23/Imp β1 (black open dots). The time-course data were fitted to an exponential function of the form *f(t)* = *f*max(1−*e*−*kt*), where *t* is time, *f(t)* is the nuclear fluorescence, *f_max_* is the endpoint of the reaction and *k* is the first-order rate constant. The fits revealed that the initial transport rate (*r = kf_max_*) of rpL23/Imp β1 was approximately three-fold higher than that of rpL23 alone.

In this paper, our major goal is to examine the role played by molecular surface charge, compared to the influence of molecular size and specific signal, in determining the transport kinetics and spatial transport routes for both passive and facilitated transport. To this end, we first employed a new imaging approach recently developed in our lab, single-point edge-excitation sub-diffraction (SPEED) microscopy (Fig. 1A), that enables the following capabilities for mapping nucleocytoplasmic transport in HeLa cells [27]–[28], [30]: 1) capture real-time fast movements of transiting molecules (typically a few milliseconds) through the native NPCs in cells with high temporal resolution (0.4 ms in SPEED microscopy, as shown in Fig. 1B and Movie S1–S6); 2) spatially distinguish and localize the individual NPCs on the NE (1–3 nm localization precision for the centroid of NPC) and simultaneously track the transiting molecules through the NPCs with high spatial resolution (particle tracking precision <10 nm for substrates moving through the NPC, as observed in Fig. 1C); and 3) obtain a 3D view of real-time spatial transport routes for the inherently 3D moving substrates in the NPC via a deconvolution algorithm (demonstrated in Fig. 1D and Movie S7–S10). Second, to specifically examine charge-dependent nucleocytoplasmic transport, we selected proper substrates of the same sizes, the same specific signals, and similar surface hydrophobicity (another factor that is suggested to affect nuclear transport), but differ only in surface charge. Here, the chosen substrates include green fluorescence protein (−7GFP, its inherent net negative surface charges is −7) and its super-charged mutations with either a positive charge of +36 (+36GFP) or a negative charge of −30 (−30GFP) in our tests (Fig. 2). Previously, these super-charged GFPs were successfully applied to study the transfection process in live cells, showing their suitability in cellular fluorescence measurements [33]–[34]. With the same molecular weight of ∼27 kDa, the same hydrodynamic size of ∼ 6 nm in diameter (determined by dynamic light scattering as shown in Fig. S1) and differing only in surface charge, the above GFP candidates are well below the cut-off size of ∼ 40 kDa (∼ 10 nm in diameter) for passive diffusion and are thus suitable for testing the effect of molecular charge on passive diffusion. Additionally, to prepare charged signal-dependent cargos for facilitated translocation, we further fused an NLS (PKKKRKV) to each of the above charged GFPs and thus obtained differently charged import cargo complexes of the NLS-GFP/transport receptor (Fig. 3 and Table 1). Finally, we tested the effect of surface charge on the nucleocytoplasmic transport of a construct of the endogenous ribosomal protein rpL23 (Fig. 4).

**Table 1:** Transport kinetics and physical dimension of transport routes for passive and facilitated nucleocytoplasmic transport.

Protein	MW (kDa)	Charge (kT/e)	D_m_ (nm)	D_p_ (nm)	L_p_ (nm)	τ (ms)	E (%)		B (%)	
							CN	NC	CN	NC
(−30)GFP	28	−30	5.9±0.7*	18.9±0.2	190±7	2.9±0.1	56±5	51±5	29±1	32±1
(−7) GFP	28	−7	6.1±1.1*	17.4±0.2	179±7	4.0±0.2	47±5	48±5	14±1	13±1
(+36) GFP	28	+36	5.8±0.9*	11.7±0.1	114±7	4.8±0.1	54±5	56±5	9±1	12±1
Impβ1	97	−48	∼9.8	–	–	5.0±0.2	52±5	47±5	22±1	22±1
(−30)GFP complex	185	−97	∼12.2	–	–	4.8±0.2	52±5	–	25±1	–
(−7)GFP complex	185	−74	∼12.2	–	–	5.5±0.1	54±5	–	21±1	–
(+36)GFP complex	185	−31	∼12.2	–	–	6.0±0.2	49±5	–	19±1	–
rpL23	16	+18	∼5.4	18.1±0.1	152±8	2.8±0.3	55±5	57±5	10±1	12±1
rpL23 complex	113	−30	∼10.3	–	–	6.0±0.1	58±5	–	28±1	–

A detailed description of determination of molecular size and dimension of transport routes are included in “Methods and Materials”. Particularly, to experimentally test whether the surface charge of GFP alters its hydrodynamic size, we have determined the size of −30GFP, −7GFP, +36GFP in solution by dynamic light scattering (*). GFP complex refers the complex of NLS-GFP/Imp α/Imp β1; rpL23 complex means the complex of rpL23/Imp β1; MW, molecular weight; D_m_, diameter of protein candidate; D_p_ and L_p_, effective diameter and length of the axial central channel; τ, measured transport time; CN, import events from cytoplasm to nucleus; NC, export events from nucleus to cytoplasm; E, transport efficiency; B, pore binding frequency.

## Results and Discussion

### The Size, not Negative Surface Charge, of Small Signal-Independent Molecules Determines their Spatial Diffusion Routes through the NPC

The first critical question is which criterion, the size or the surface charge of signal-independent small molecules, plays a dominant role in determining their spatial passive diffusion routes in the NPC. The hydrogel-like meshwork model suggests that numerous holes in the mesh can serve as passive-diffusion channels for small molecules such as GFPs [19]. Because most FG Nups have net positive charges, the holes (<2.6 nm in diameter) proposed in the FG-filament meshwork could form many positively charged micro-environments for passengers [35], which would attract negatively charged or repel positively charged molecules [17]. Specifically, for the tested charged GFP candidates (−30GFP, −7GFP and +36GFP), the prediction would be that only negatively charged GFPs could diffuse through the NPCs, whereas the positively charged GFPs would be rejected at their entries into the NPC. In contrast, single-channel models, including the oil-spaghetti model [20], the reduction of dimensionality (ROD) model [21], and the self-regulated viscous channel (SERVICE) model [27], propose that a central axial channel [10–17 nm in diameter) acts as the major diffusion conduit for small molecules. The SERVICE model further suggests that the FG filaments concentrated at the periphery around the central channel provide major binding sites for transport receptors and their facilitated cargo complexes [27]. Given that the effective distance of electrostatic interactions between charged particles is approximately 1 nm [17], such a configuration of the relative big central channel and the peripheral concentrated FG filaments in the NPC suggests that small molecules, whether negatively or positively charged, should be able to diffuse through the central channel.

To test the above predicted diffusion routes for small charged molecules from different transport models, we measured the 3D transport routes for Alexa Fluor 647-labeled charged GFPs (−30GFP, −7GFP, +36GFP) through the GFP-labeled NPCs by combining SPEED microscopy and the deconvolution algorithm (Fig. 2 and Movie S7). By SPEED microscopy, approximately 5,000 spatial locations for each charged GFP were collected from 110–750 single molecule nucleocytoplasmic transport events in 10 NPCs of 10 cells. The obtained spatial locations revealed the 2D spatial distributions of each charged GFP in the NPC, in which all GFPs appeared to concentrate in a central axial region at the central scaffold of the NPC, but whether they diffuse through a single channel or multiple channels was not clear. As their corresponding 3D spatial probability density maps were obtained, it became clear that all GFPs passively diffused through a central axial channel in the NPC regardless of their charge (Fig. 2-IV). The above results agree very well with the 3D passive diffusion paths that we previously obtained for other small molecules, including small organic dyes, small dextran molecules and a few small endogenous proteins [27]. In contrast, we found that GFP dimers, by having a net negative surface charge of −14, cannot passively diffuse through the NPC due to their large size of approximately 61 kDa [27]. Therefore, these measurements indicate that the size of GFPs, rather than their surface charge, predominantly determines the spatial location of their passive diffusion routes. Additionally, these observations support the prediction of the SERVICE model that charged small molecules, whatever their charges, passively diffuse through a central axial channel in the NPC.

Meanwhile, we also found that the lengths and the diameters of the effective diffusion routes for the positively charged GFPs were shorter and smaller than those of their negatively charged partners (Fig. 2-IV). In particular, we observed a decrease in the effective length distribution from 190±7 nm to 114±2 nm (with the major difference in length on the nucleoplasmic side of the NPC) and a reduction in the effective width distribution from 18.9±0.2 nm to 11.7±0.1 nm as the GFP’s charge is increased from −30 to +36. Such changes suggest that molecular charge, though not a dominant factor in deciding the spatial location of passive diffusion route, plays a noticeable role in shaping the dimension of the central diffusion path through the electrostatic interactions between the charged GFPs and the charged Nups of the NPC. It is possible that attraction between −30GFP and the peripheral positively net charged FG-Nup domains could induce the negatively charged GFPs to move slightly off the pore axis and towards the periphery, as well as decelerate their movements as they exit the nuclear pore and thus generate a wider distribution at the cross-section of the NPC and a longer diffusion path along the pore axis. Conversely, for +36GFPs, the repulsive electrostatic forces between the same positively charged transiting molecules and FG Nups could behave in exactly the opposite manner: the net positively charged FG Nups at the periphery could confine these positively charged molecules around the pore axis as they diffuse through the NPC and accelerate them out of nuclear pore as they exit. Thus, a shorter and narrower effective diffusion pathway for +36GFPs is generated. In contrast to the previously proposed model [17], the net positive charge of the FG-barrier does not prevent the positively charged molecules such as +36GFPs, as long as the molecules are smaller than a cut-off limit (∼ 40 kDa), from diffusing through the NPC. The determined length and width of the effective transport path of −7GFPs are between those of the supercharged versions (Fig. 2B), as expected. The following measurements of the transport kinetics for charged GFPs, including transport time, transport efficiency and pore binding frequency, will provide more insight into the charge effect on passive diffusion through the NPC.

### Negative Molecular Surface Charge Enhances Higher Nuclear Pore Binding Probability and Shorter Transport Time for Passive Diffusion through the NPC

Using SPEED microscopy, we first localized individual GFP-labeled NPCs on the NE and then tracked single Alexa647 Fluor labeled molecules moving through these NPCs (Movie S1–S2). Superimposing the obtained trajectories of single in-transit molecules and the localized centroid of the NPC (Fig. 1C and Fig. S1), we can readily determine how many molecules moving from the cytoplasmic or the nuclear side bind to the NPC per second (pore binding frequency), how long the molecules take to travel through the NPC (transport time) and the percentage of molecules that successfully complete their transport through the NPC (transport efficiency). As shown in Table 1, the major differences among the three charged GFP candidates are their pore binding frequencies and transport times. We found that the pore binding frequency of −30GFP is approximately two-fold and three-fold of the pore binding frequency of −7GFP and +36GFP, respectively. Also, the transport times for charged GFPs were determined to be 2.9±0.1 ms for −30GFP, 4.0±0.2 ms for −7GFP and 4.8±0.1 ms for +36GFP (Table 1). However, as soon as these differently charged GFPs enter the NPC, their transport efficiencies (both the import efficiencies and the export efficiencies) are almost the same. These results clearly suggest that the molecules with higher negative charges have greater opportunity to initiate their interactions with the NPC and higher initial velocity to start their diffusion through the NPC due to stronger attractions with the positively charged FG Nups of the NPC, but they do not have higher priority for traversing the NPC than the less negatively charged or positively charged molecules once they move into the NPC.

### Transport Kinetics and 3D Transport Routes of Facilitated Translocation of Various Charged Cargos

Almost all currently known nucleocytoplasmic transport receptors, including importins and exportins, are reported to have net negative surface charges [17]. In nucleocytoplasmic facilitated transport, these negatively charged transport receptors recognize cargo proteins that carry specific signals and chaperone them through the NPC by directly conducting hydrophobic interactions with the net positively charged FG Nups [14]–[16]. In addition to the established major role of hydrophobic interactions, the electrostatic interactions between transport receptors and FG Nups were recently suggested to dramatically affect facilitated transport as well [17]. However, the surface charges of cargo proteins in yeast or human cells vary significantly from −30 to +30 [17]. How the surface charges of cargo proteins could affect the transport kinetics and spatial transport routes of transport receptor-facilitated translocation is also of great interest and the aim of our tests.

Importin β1, a major importin with a net negative charge of −47, recognizes NLS-tagged cargo proteins via importin α (another importin with a net negative charge of −20) to form a cargo complex of Imp β1/Imp α/cargo in the cytoplasm [36]. During facilitated import, Imp β1 directly interacts with FG Nups and drives the cargo complex to diffuse through the FG-filament-filled periphery around the central axial passive diffusion channel of the NPC within approximately 5–10 ms [30], [36], [37]–[39]. Finally, a high concentration of RanGTP in the nucleus enhances the dissociation of the cargo complexes in the nucleus whereupon Imp β1 and Imp α diffuse back to the cytoplasm for the next cycle of facilitated translocation [16], [40]. Here, we use the Imp β1/Imp α/cargo system as a model system to study the effect of the cargo’s charge on facilitated translocation by determining the transport kinetics and the 3D transport route of Imp β1 when it forms complexes with differently charged cargos. Given that the net surface charge of endogenous cargo proteins varies mainly between −30 to +30, in our experiments, we fused NLS (PKKKRKV) to charged GFPs to produce charged signal-dependent cargos of NLS-(−30GFP), NLS-(−7GFP) and NLS-(+36GFP) to test the charge effect on facilitated translocation. These designed candidates are of the same sizes and similar surface hydrophobicity, but differ only in charge (Table 1).

By employing single-particle tracking by SPEED microscopy and the 3D deconvolution algorithms, we obtained the transport kinetics and 3D transport routes for the facilitated translocation of the above three cargo complexes (Fig. 3, Fig. S2 and Table 1). Although the net charge of cargo NLS-GFP changed from −30 to +36 and the net charge of cargo complexes Imp β1/Imp α/NLS-GFP changed from −97 to −31, we found that the cargo’s charges did not cause major changes in either the transport kinetics or the 3D transport routes of Imp β1-assisted facilitated translocations. In detail, regardless of the charge of NLS-GFP, Imp β1/Imp α/NLS-GFP complexes always go through the NPC through the periphery around the central axial channel with transport times of approximately 5–6 ms, similar to that of Imp β1 alone (Fig. 3 and Table 1). However, in contrast to the assumption that more negative cargo complexes would receive more electrostatic attraction from the FG Nups anchored on the pore wall, we found that the complex of Imp β1/Imp α/−30GFP with the highest negative charges stays farther away from the pore wall and generates a narrower unoccupied central channel than the other two in-transit complexes (Fig. 3B). Rather than electrostatic attraction between oppositely charged objects, it seems that there is the electrostatic repulsion on Imp β1/Imp α/−30GFP from the pore wall. Previously it was reported that concentrated Imp β1 molecules (up to 100 Imp β1s) could bind to FG Nups in a single NPC [48]–[49]. Although the majority of the soluble molecules in the cytoplasm are supposed to be washed away in permeabilized cells [50], there might still be some residue Imp β1s and/or other transport receptors binding to FG Nups in the NPCs. When Imp β1/GFP complexes come for interacting with the FG Nups in the NPCs, those bound residue negatively charged transport receptors might produce the electrostatic repulsion for Imp β1/GFP complexes, which could affect the bindings of these complexes with the available FG Nups and also push them away from the pore wall. Compared to the other two candidates, the effect of electrostatic repulsion could be more obvious for Imp β1/Imp α/−30GFP since it has the highest negative charges.

Overall, the above observations indicate that Imp β1-driven hydrophobic interactions with the FG Nups dominate the facilitated translocation of these charged cargo complexes functionally and spatially. Meanwhile, some changes observed in the diameter of the central channel, the transport time and the pore binding frequency for these charged candidates also suggest a minor role of electrostatic interactions between import cargo complexes and the FG Nups in facilitated transport (Fig. 3 and Table 1).

### Passive and Facilitated Import of a Charged Endogenous Protein through the NPC

A critical remaining question was whether the above conclusions on passive and facilitated transport based on experiments using exogenous charged GFPs would be applicable to endogenous proteins. To answer this question, we adapted a ribosomal protein rpL23 (also known as 60S ribosomal protein L17) to test the above conclusions. rpL23, a component of the 60S subunit and one of the approximately 80 structurally distinct endogenous proteins in human ribosomes, is reported to be actively imported from the cytoplasm to the nucleus to maintain the structural integrity of the ribosome [41]–[44], but its detailed transport kinetics and 3D transport route through the native NPCs remain unclear. rpL23 possesses a net positive surface charge of approximately +18, a molecular weight of approximately 19 kDa and an IBB domain that enables it to form a transit complex with Imp β1. Interestingly, the above features could generate conflicting predictions for the transport mode of rpL23 through the NPC, depending on whether its transport through the nuclear pore is dominated by its size, its specific signal or its surface charge. In particular, its size, which is below the cut-off size for passive diffusion, suggests the possibility that rpL23 would passively diffuse through the NPC; in contrast, the IBB domain that rpL23 carries suggests that it could follow an Imp β1-faciliated translocation pathway through the NPC. Given the high net positive surface charge of rpL23, it may even be repelled from entering the NPC according to the previously proposed model [17]. However, the results that we have obtained for charged GFPs predict that rpL23 alone is small enough to passively diffuse through the axial central channel in the NPC, but with a low pore binding frequency because of its positive surface charge. Moreover, when rpL23 forms a complex with Imp β1, the rpL23/Imp β1 complex would conduct facilitated transport and its 3D pathway would follow Imp β1’s route in the periphery, distinct from the central axial channel for passive diffusion in the NPC.

To test the above predictions and clarify the nuclear transport mode of rpL23, we tracked labeled rpL23 through the native NPC at the single-molecule level with or without binding to Imp β1 in a permeabilized HeLa cell system using SPEED microscopy (Fig. S3). In Fig. 4A, the 3D mapping of transport routes clearly shows that rpL23 alone passively diffused through an axial central channel in the NPC with a relatively low pore binding frequency (Table 1) or alternatively followed Imp β1-driven facilitated translocation through the peripheral region around the central channel when forming a cargo complex with Imp β1 (Fig. 4B), but it was never completely repelled from entering the NPC (Table 1). The experimental data appear to have confirmed our predictions of the transport mode for rpL23 with or without forming a cargo complex with Imp β1, and they further indicated that the IBB domain of rpL23 plays a dominant role in its nuclear import routes compared to its size and surface charge when in the presence of Imp β1.

Given the distinct spatial locations of transport routes between rpL23 alone and the rpL23/Imp β1 complex, a natural question is which path would enable a more efficient import of rpL23 from the cytoplasm to the nucleus under physiological conditions. To answer this question, we compared the time-courses of net nuclear accumulation of rpL23 with or without the presence of Imp β1 in permeabilized HeLa cells. By epi-fluorescence microscopy, we found that the facilitated transport of rpL23/Imp β1 complex generated approximately three-fold higher initial transport rate and about six-fold greater final nuclear accumulation of rpL23 than the passive diffusion of rpL23 alone (Figure 4C). This result is consistent with our previous observations of net nuclear accumulation of labeled signal-dependent cargo proteins in living cells [38].

The transport kinetics obtained at a single-molecule level for both rpL23 alone and the rpL23-Imp β1 complex further suggest that the pore binding frequency, not the transport time and efficiency, becomes the critical step in determining the initial transport rate for either the passive diffusion of rpL23 or the facilitated transport of rpL23-Imp β1 complexes through the NPC (Fig. 4 and Table 1). When incubating the same concentrations of rpL23 with the permeabilized cells, approximately ten rpL23 alone and twenty eight rpL23/Imp β1 complexes were observed to interact with each NPC per second, and approximately half of them completed nuclear transport. The approximately three-fold difference in pore binding frequencies combined with the same transport efficiencies resulted in an almost three-fold difference in the initial transport rates for passive and facilitated transports as the same as that observed at bulk experiments (Fig. 4C), which was independent of their transport times of 2.8±0.3 ms and 6.0±0.1 ms. The observed higher frequency of pore binding for rpL23/Imp β1 compared to rpL23 alone may be due to the combined influence of both the hydrophobic and the electrostatic interactions between Imp β1 and FG Nups.

Moreover, in permeabilized cells, the intact nucleus, together with a controlled cytoplasmic environment, provides a greatly simplified transport system that allows us to elucidate the complicated nuclear transport step by step [27]–[31], [50]. But whether the results obtained in permeabilized cells could mimic nuclear transport in live cells always needs to be tested. Previous studies have shown similar nucleocytoplasmic transport kinetics for cargo-free and cargo-bound transport receptors in permeabilized and live cells [38]–[39]. Here we further explored whether the measurements on nuclear import kinetics of rpL23 in permeabilized cells could hint its nuclear import in live cells. In detail, we genetically engineered rpL23-mCherry in live GFP-POM121 HeLa cells and measured the nuclear import time for rpL23-mCherry, obtaining two distinct nuclear import times for rpL23-mCherry in live cells, which are 2.1±0.5 ms and 6.0±0.6 ms (Figure S4 and Table 1). These times agree well with the measured times for rpL23 alone and rpL23/Imp β1 complex in permeabilized cells (Table 1). This agreement, consistent with the previous tests [38]–[39], confirmed that the experiments conducted in permeabilized cells provide great insights into nuclear transport mechanism in live cells.

## Conclusion

**Figure 5:**
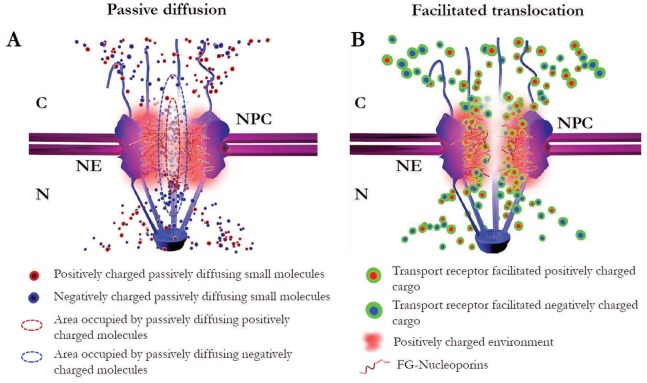
Summary of the charge effect on nucleocytoplasmic transport. (A) Charge effect on passive diffusion. Small positively charged molecules (<40 kDa, red dots) permeate the NPC through the central channel just as small negatively charged (blue dots), but with a shorter and narrower route. (B) Charge effect on facilitated transport. Regardless of the cargo’s charge, transiting cargo complexes (transport receptor/cargo) follow the pathways of the transport receptor through the FG filamentous regions at the periphery around the central channel.

In conclusion, we characterized the charge-dependent transport kinetics and 3D transport routes for both passive and facilitated transport by employing the newly developed SPEED microscopy and the 3D deconvolution algorithms in this paper. Our major goal is to determine which property, the size, the specific signal or the surface charge of transiting molecules, plays the dominant role in determining their transport kinetics and their 3D transport routes through the NPC during both passive and facilitated nucleocytoplasmic transport. Our major findings are summarized as follows. First, for passive diffusion of signal-independent small molecules, the size, not the surface charge, determines the 3D spatial location of their paths through the NPC. Our experimental data clearly show that small molecules, whatever their charge, diffuse through an axial central channel in the NPC. Meanwhile, the molecular charges slightly affect the dimension of transport paths and the diffusion times through the NPC. Second, for charged cargo molecules with specific signals that can be recognized by transport receptors, hydrophobic interactions, not electrostatic interactions, between transport receptors and FG Nups in the NPC play a dominant role in determining the transport kinetics and the 3D spatial transport routes during their facilitated translocation. Our data indicated that the spatial pathways of various charged cargo molecules always follow their transport receptors’ paths at the periphery surrounding the axial central channel in the NPC, though the surface charge of cargo molecules slightly affects the transport kinetics and routes of cargo-transport receptor complexes through the NPC. Third, the 3D spatial locations of charged small molecules, transport receptors, and cargo complexes also enabled us to plot a comprehensive map of interactions regarding the combined effect of the molecular size, the carried specific signal and the surface charge in the NPC. As shown in Fig. 5, our data suggest that there is an inhomogeneous spatial distribution of interactions between transiting molecules and FG Nups in the NPC. In particular, the filaments of FG Nups may form net positively charged regions at the periphery around the inner wall of NPC, while the axial central channel filled with very sparse FG filaments provides an almost neutral conduit for passive diffusion. Small signal-independent molecules, whatever their charge, passively diffuse through the neutral central channel, and the transport-receptor-facilitated signal-dependent charged cargo molecules (either positive or negative) go through the peripheral positively charged regions by hydrophobic-interaction-driven paths.

The results of several previous investigations have suggested that the surface hydrophobicity of large cargo proteins (>40 kDa) itself, independent of transport receptors, could enable successful nucleocytoplasmic transport of large cargos [45]–[47]. However, whether there is a distinct principle or a similar mechanism between cargo-surface-hydrophobicity-driven nuclear transport and transport-receptor-driven cargo translocation remains unknown. The research strategies we employed in this paper could be applied to further investigate the role of molecular hydrophobicity on nucleocytoplasmic transport.

## Materials and Methods

### Cell Culture and Transport Condition

A stable HeLa cell line with genetically GFP-fused POM 121 (a Nup located at the middle plane of the central scaffold region of the NPC) was used in our experiments. Freshly split HeLa cells were grown on glass coverslips overnight at 37°C with 5% CO_2_ in Dulbecco’s modified Eagle’s medium (D-MEM) supplemented with 4.5 g/L glucose, 862 mg/L GlutaMAX-I, 15 mg/mL phenol red, 100 U/mL penicillin, 100 µg/mL streptomycin, and 10% (v/v) FBS. For single molecule measurements of nuclear import of rpL23 in live cells, we have transferred rpL23-mCherry and controlled its expression level to be low enough for single molecule imaging in live GFP-POM121 HeLa cells. For single molecule measurements of labeled proteins in permeabilized HeLa cells, the cell confluency on the cover slip was controlled not to exceed 50% to provide a good freedom for exchange of buffers and nuclear transport of proteins of interest. Flow chambers on the coverslip were constructed by adding a top cover slip together with two lines of silicone grease as spacers. Then, the bottom of the slide was cleaned with a cotton-tipped swab, twice with water, and then twice with 100% ethanol. Once the slide was mounted on the microscope, the cells were washed with transport buffer (20 mM HEPES, 110 mM KOAc, 5 mM NaOAc, 2mM MgOAc, 1 mM EGTA, 1 mM DTT, pH 7.3) first. The cells then were permeabilized for approximately 2 minutes with 2×25 µL of 40 µg/mL digitonin in transport buffer and finally washed with 4×25 µL transport buffer supplemented with 1.5% polyvinylpyrrolidone (PVP; 360 kDa). PVP was included in all import buffer solutions after permeabilization to prevent osmotic swelling of nuclei. The intact nucleus, together with a controlled cytoplasmic environment, provides a greatly simplified transport system that allows us elucidate the complicated nuclear transport.

For the facilitated nuclear import of NLS-GFP at single molecule level, 1 nM Alexa Fluor 647 labeled GFP molecules were incubated with permeabilized cells by adding transport cofactors of 0.5 µM Imp β1, 0.5 µM Imp α, 1 mM GTP, 2 µM Ran and 1 µM NTF2. For the efficient nuclear import of rpL23, all the same cofactors except Imp α were used for single molecule measures of 1 nM Alexa Fluor 647 labeled rpL23 and bulk experiments of 0.2 µM labeled rpL23. The labeling was performed on cysteins available on the protein surface in ratio one dye per protein. His tag on the protein was not removed as it’s considered not to affect the functionality of the protein. For passive diffusion of signal-independent small molecules, only substrate without transport cofactors was used.

### Protein Purification and Labeling

−30 GFP, −7 GFP and +36 GFP were expressed with N-terminal 6xHis-tags (plasmids were kindly provided by Dr. D. Liu, Howard Hughes Medical Institute, Harvard University). Proteins were overexpressed in *Escherichia coli* BL21(λDE3) strain by 1 mM IPTG induction. Branson Sonic Dismembrator 550 was used 2 times at 5 minutes with 50% duty cycle and output 3 to break up the cells. The extract then was purified by Superflow (Qiagen, Valencia, CA), MonoQ, and Superdex 200 (Amersham Pharmacia) chromatography. The three chromatographic steps were necessary to yield a single band by Coomassie-stained polyacrylamide gel electrophoresis. All transport cofactors were purified as described in our previous reports [27]–[31].

To study the charge effect on the facilitated nuclear transport and assure the formation of the nuclear transport complex, −30 GFP, −7GFP and +36 GFP were engineered to carry NLS sequence usually consisting of simple five amino acids peptide PKKKRK. The sequence encoding for PKKKRK peptide was synthesized with two unique restrictions sites XbaI and NcoI on either ends and put in pUC57 vector with Amp gene. The fragment was then subcloned into regions between XbaI and NcoI on pET-GFP-NEG30 (−30 GFP), pET-GFP-NEG7 (−7 GFP) and pET-GFP-POS36 (+36 GFP) to assure a proper expression of −30 NLS-GFP, −7 NLS-GFP and +36 NLS-GFP. The final coding regions were confirmed by DNA sequencing. rpL23 was expressed in *Escherichia coli* and purified as mentioned above.

The solvent-accessible cysteines on GFPs and rpL23 were labeled with 20-fold molar excess Alexa Fluor 647 maleimide dye (Invitrogen) for 2 h at room temperature in 50 mM sodium phosphate, 150 mM NaCl, pH 7.5. Reaction was quenched with β-mercaptoethanol, and the products were dialyzed to remove the free dyes. The labeling ratio is 1 dye per GFP or rpL23.

### Instrumentation

The SPEED microscope setup has been extensively described in previous publications [27], [30]. Briefly, it consists of an Olympus IX81 with a 1.4 NA 100× oil-immersion apochromatic objective (UPLSAPO 100X, Olympus), a 35 mW 633 nm He-Ne laser (Melles Griot), a 120 mW ArKr tunable ion laser (Melles Griot), an on-chip multiplication gain charge-coupled device camera (Cascade 128+, Roper Scientific) and the Slidebook software package (Intelligent Imaging Innovations) for data acquisition and processing. Cascade 128+ (128×128 pixels) was used to monitor the fast nuclear import process.

An optical chopper (Newport) was used to generate an on-off mode of laser excitation. GFP and Alexa Fluor 647 fluorescence were excited by 488 nm and 633 nm lasers, respectively. The two lasers were combined by an optical filter (FFF555/646 Di01, Semrock), collimated and focused into an overlapped illumination volume in the focal plane. The green and red fluorescence emissions were collected by the same objective, filtered by a dichroic filter (Di01- R405/488/561/635-25×36, Semrock) and an emission filter (NF01- 405/488/561/635-25×5.0, Semrock) and imaged by an identical CCD camera. The system error of alignment between red and green fluorescence channels is 3.0±0.1 nm, determined by measuring 230 immobile Alexa Fluor 647-labeled GFP fluorescent molecules on the surface of a cover-slip.

### Determination of Molecular Diameter

The molecular diameters have been measured experimentally by gel filtration, size exclusion column and dynamic light scattering. Additionally, contribution of labeled dyes on the substrate for the final size of substrate was determined by the relationship between diameter (*d*) and molecular weight (*MW*) with (*w*) or without (*o*) labeled dyes as 
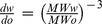
 Particularly, to experimentally test whether the surface charge of GFP alters its hydrodynamic size, we have determined the hydrodynamic size of −30GFP, −7GFP, +36GFP in solution by dynamic light scattering. In detail, 500 µL of 1 mg/mL of each candidate in PBS buffer was loaded onto Malvern Zetasizer Nano DLS and the size distribution by intensity, mass and volume were measured multiple times and averaged (Fig. S1). The average sizes were determined to be 5.9±0.7 nm, 6.1±1.1 nm and 5.8±0.9 nm for −30GFP, −7GFP and +36GFP, which are almost the same given the error bars. Therefore, the above results revealed that the difference surface charges of the above GFP candidates have negligible effect in altering their hydrodynamic sizes.

### Determination of Effective Transport Routes of In-Transit Molecules in the NPC

The obtained 3D spatial probability density of in-transit molecules in the NPC is normalized by the highest density along the transport path. The densities which are within 95% of the highest one are defined as the effective transport route for in-transit molecules through the NPC. Only the effective path is shown in the 3D views for all in-transit substrates.

### Localization and Orientation of a Single NPC

First, the position of the nuclear envelope was determined by epifluorescence microscopy with engaging a HBO mercury lamp with a set of GFP filters to take a picture of the NE. The pixel intensities within a row or column are approximately perpendicular to the NE were fit with Gaussian. The peak positions of the Gaussian for a particular set of pixel intensities was considered the NE position for that row and column. The peak positions of a series of such Gaussians were then fit with a second-degree polynomial, yielding the path of the NE within the entire image. To select a single NPC oriented perpendicularly to the NE on the equator of the nucleus and to the y direction of the Cartesian coordinates (x, y) in the CCD camera: first choose a fluorescent NPC on the equator of the nucleus such that the tangent of the NE at the location of this NPC should be parallel to the y direction of the Cartesian coordinates (x, y) in the CCD camera; and second examine the ratio of Gaussian widths in the long and short axes of the chosen GFP-NPC fluorescence spot. The ratio needs to fall between 1.74 and 1.82. Within this range, an illuminated NPC only has a free angle of 1.4° to the perpendicular direction to the NE [30].

### Localization Precision for Isolated Fluorescent Spots

To determine the localization precision for immobile molecules, their fluorescent spots were fitted to 2D symmetrical or elliptical Gaussian functions, and the localization precision was determined by the standard deviation of multiple measurements of the central point. However, for moving molecules, the fluorescent spot was fitted to 2D elliptical Gaussian functions, but the localization precision (σ) was determined by an algorithm of σ =  
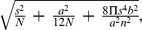
 where N is the number of collected photons, a is the effective pixel size of the detector, b is the standard deviation of the background in photons per pixel, and s is the standard deviation of the point spread function. In our measurements, the localization precision is approximately 9–12 nm for the immobile molecules and 10–13 nm for the moving molecules when around 1,100 photons were collected from the targeted molecule, and the signal to noise (S/N) ratio was ≈ 11 determined as 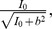 where I_0_ is the maximum signal in photons of the pixel in the detection point spread function.

### Determination of Electrostatic Potentials

To calculate and visualize electrostatic surface potentials of the candidate molecules, an adaptive poison-boltzmann solver APBS tool [51] was applied to three dimensional protein structures obtained from RSCB PDB data base. Protein structures had to be prepared for APBS calculations by reconstructing missing heavy atoms, addition of hydrogens, removing excessive water molecules and designating atomic charges and radii. For that purpose PDB2PQR tool freely accessible at http://nbcr-222.ucsd.edu/pdb2pqr_1.8/was applied with subsequent generation of PQR files or electrostatic potential maps [52]. To visualize the obtained electrostatic maps for each protein structure, Chimera software suite was utilized [53].

### Transport Time

To determine the transport time of the fluorescent in-transit molecules through the NPC, video frames were analyzed in Metamorph software. The videos of the transiting molecules were merged with the localized NPC. The transport time was then determined based on the localization of the cargo molecules (single-molecule trajectories) relatively to the centroid of NPC. A complete transport event included the total number of frames from where the molecule enters the NPC starting from one compartment (either cytoplasm or nucleus) and exits to the other compartment. Cytoplasm to nucleus and cytoplasm to cytoplasm events were counted as import events, and nucleus to cytoplasm and nucleus to nucleus as export events. All measured times were used to generate the histograms of time versus occurrence frequency. The histograms were finally fitted with an exponential decay function to get an averaged time for import or export events.

### Transport Efficiency

Transport events were legitimate to be considered in analysis, when a trajectory of a transiting molecule showed that it entered the 100-nm proximal area of the NPC from either cytoplasmic or nucleoplasmic side. Two types of translocation events could be distinguished: complete transport events, when a cargo complex traversed the NPC from one compartment to another one; and abortive events, when a cargo entered the NPC, interacted with it and then turned back and exited to the original compartment. The former consists of trajectories for which the first and last points are at least 100 nm from the centroid of NPC, and a line between these points crosses the NE. The latter consists of trajectories for which the first and last points are at least 100 nm away from the centroid of NPC and both are on either cytoplasmic or nucleoplasmic side. There also was a third class of crossing events, when the trajectories did not fall into either the complete events category or abortive events category, because the exit compartment was not clear and therefore they were not considered during analysis. The import/export efficiency was derived from the number of complete events divided by the sum of numbers of both complete and abortive events and is reported as a percentage.

### Pore Binding Frequency

Pore binding frequency was defined as the total number of complete and abortive events that originated on either cytoplasmic or nucleoplasmic side divided by the total time of the video and the number of nuclear pores that these events were obtained from.

### Deconvolution

The deconvolution process used to obtain the 3D spatial distribution of interaction sites between transiting molecules and the NPC was the same as that described in previous publications [27], [30].

### Sequences of GFP Candidates

−**30 GFP.** MGHHHHHHGGASKGEELFDGVVPILVELDGDVNGHEFSVRGEGEGDATEGELTLKFICTTGELPVPWPTLVTTLTYGVQCFSDYPDHMDQHDFFKSAMPEGYVQERTISFKDDGTYKTRAEVKFEGDTLVNRIELKGIDFKEDGNILGHKLEYNFNSHDVYITADKQENGIKAEFEIRHNVEDGSVQLADHYQQNTPIGDGPVLLPDDHYLSTESALSKDPNEDRDHMVLLEFV AAGIDHGMDELYK.

−**7GFP.** MGHHHHHHGGASKGEELFTGVVPILVELDGDVNGHKFSVRGEGEGDATNGKLTLKFICTTGKLPVPWPTLVTTLTYGVQCFSRYPDHMKQHDFFKSAMPEGYVQERTISFKDDGTYKTRAEVKFEGDTLVNRIELKGIDFKEDGNILGHKLEYNFNSHNVYITADKQKNGIKANFKIRHNVEDGSVQLADHYQQNTPIGDGPVLLPDNHYLSTQSALSKDPNEKRDHMVLLEFVTAAGITHGMDELYK.

**+36 GFP.** MGHHHHHHGGASKGERLFRGKVPILVELKGDVNGHKFSVRGKGKGDATRGKLTLKFICTTGKLPVPWPTLVTTLTYGVQCFSRYPKHMKRHDFFKSAMPKGYVQERTISFKKDGKYKTRAEVKFEGRTLVNRIKLKGRDFKEKGNILGHKLRYNFNSHKVYITADKRKNGIKAKFKIRHNVKDGSVQLADHYQQNTPIGRGPVLLPRNHYLSTRSKLSKDPKEKRDHMVLLEFVTAAGIKHGRDERYK.

### Standard Error

Experimental measurements are reported as means ± standard errors of the mean unless otherwise noted.

## Supporting Information

Figure S1 Determination of the hydrodynamic sizes of all GFP candidates by dynamic light scatting. 500 µL of 1 mg/mL of each candidate in PBS buffer was loaded onto Malvern Zetasizer Nano DLS for the measurements. The size distribution by intensity, mass and volume were measured multiple times and averaged. The average sizes were determined to be 5.9±0.7 nm, 6.1±1.1 nm and 5.8±0.9 nm for −30GFP (blue), −7GFP (green) and +36GFP (red). The number in the bracket is the diameter with s. d. in nanometer.
(TIF)

Figure S2 Typical single-molecule trajectories of nucleocytoplasmic transport events of signal-independent and signal-dependent GFPs obtained by SPEED microscopy. (A) A typical nuclear import event of supercharged −30GFP molecules from the cytoplasm to the nucleus. First, a single GFP-NPC (green spot) was visualized in the illumination volume. Then, a single fluorescent −30GFP molecule (red spot) entered the illumination volume, started in the cytoplasm (C), interacted with the NPC and entered the nucleus (N). Numbers denote time in millisecond. Scale bar: 1 µm. (B) Single-molecule trajectories of the import event in A. Based on the centroid (red dot) and the dimensions of the NPC, the spatial locations of Imp β1 molecule from 0.8 ms to 3.6 ms was within the NPC. (C and D) Individual video frames and the corresponding single-molecule trajectories of a typical export event for −30GFP. (E and F) Individual video frames and the corresponding single-molecule trajectories of a typical import event for the −30GFP import cargo complex.
(TIF)

Figure S3 Typical single-molecule trajectories of nucleocytoplasmic transport events of rpL23 obtained by SPEED microscopy. (A and B) Individual video frames and single-molecule trajectories for typical import event for native ribosomal protein rpL23. (C and D) Individual frames and the corresponding single-molecule trajectories of a typical export even for rpL23. (E and F) Individual frames and trajectories for a typical import event for the import complex rpL23-Imp β1.
(TIF)

Figure S4 Nuclear import times of rpL23-mCherry in live HeLa cells. Histogram of 237 import events for rpL23-mCherry in live cells is fit well by a double exponential decay function generating two distinct import times of 2.1±0.5 ms and 6.0±0.6 ms.
(TIF)

Movie S1 This movie shows a typical import diffusion event of −30GFP from the cytoplasm to the nucleus in a eukaryotic cell. Individual frames of this movie are shown in Fig. S1A. Pixels are 240-nm square; each frame was acquired in 400 µs; and the playback speed is 2500X slower than real time. The compartment on the right side of the NPC (the green fluorescent spot) is the cytoplasm (C), and the left side is the nucleus (N). The −30GFP molecule (the red fluorescent spot) starts in the cytoplasm, interacts with the NPC and ends in the nucleus. Individual frames of this movie are shown in Fig. S1A.
(MOV)

Movie S2 This movie shows a typical diffusion export event of −30GFP from the nucleus to the cytoplasm in a eukaryotic cell. The acquisition time and playing speed are the same as those of Movie S1. The −30GFP molecule (the red fluorescent spot) starts in the nucleus (N), interacts with the NPC (the green fluorescent spot) and ends in the cytoplasm (C). Individual frames of this movie are shown in Fig. S1C.
(MOV)

Movie S3 This movie shows a typical import event of cargo complex Imp α/Imp β1/NLS-(−30) GFP from the cytoplasm to the nucleus in a eukaryotic cell. The acquisition time and playing speed are the same as those of Movie S1. The cargo complex molecule (the red fluorescent spot) starts from the cytoplasm (C), interacts with the NPC (the green fluorescent spot) and ends in the nucleus (N). Individual frames of this movie are shown in Fig. S1E.
(MOV)

Movie S4 This movie shows a typical import diffusion event of rpL23 from the cytoplasm to the nucleus in a eukaryotic cell. The acquisition time and playing speed are the same as those of Movie S1. The rpL23 molecule (the red fluorescent spot) starts from the cytoplasm (C), diffuses through the NPC (the green fluorescent spot) and ends in the nucleus (N). Individual frames of this movie are shown in Fig. 4A.
(MOV)

Movie S5 This movie shows a typical export diffusion event of rpL23 from the nucleus to the cytoplasm in a eukaryotic cell. The acquisition time and playing speed are the same as those of Movie S1. The rpL23 molecule (the red fluorescent spot) starts from the nucleus (N), diffuses through the NPC (the green fluorescent spot), and ends in the cytoplasm (C). Individual frames of this movie are shown in Fig. S4C.
(MOV)

Movie S6 This movie shows a typical import event of cargo complex Imp β1/rpL23 from the cytoplasm to the nucleus in a eukaryotic cell. The acquisition time and playing speed are the same as those of Movie S1. The cargo complex molecule (the red fluorescent spot) starts from the cytoplasm (C), interacts with the NPC (the green fluorescent spot) and ends in the nucleus (N). Individual frames of this movie are shown in Fig. S4E.
(MOV)

Movie S7 This movie shows a cut-away view of the 3D diffusion pathway for −30GFP through the NPC (red cloud; brighter color indicates higher spatial density) superimposed on the NPC architecture (blue). Spatial dimension of effective transport route is marked in nanometer. The 3D views of the effective diffusion routes for −7GFP and +36GFP are similar as shown here for −30GFP but with different dimensions as indicated in Fig. 2. C, the cytoplasmic side of the NPC. N, the nucleoplasmic side of the NPC.
(MOV)

Movie S8 This movie shows a cut-away view of the 3D import pathway of cargo complex −30GFP/Imp α/Imp β1 through the NPC (green cloud; brighter color indicates higher spatial density ) superimposed on the NPC architecture (blue). Spatial dimension of effective transport route is marked in nanometer. C, the cytoplasmic side of the NPC. N, the nucleoplasmic side of the NPC.
(MOV)

Movie S9 This movie shows a cut-away view of the 3D diffusion pathway for native ribosomal protein rpL23 through the NPC (red cloud; brighter color indicates higher spatial density) superimposed on the NPC architecture (blue). Spatial dimension of effective transport route is marked in nanometer. C, the cytoplasmic side of the NPC. N, the nucleoplasmic side of the NPC.
(MOV)

Movie S10 This movie shows a cut-away view of the 3D import pathway of cargo complex rpl23/Imp β1 through the NPC (green cloud; brighter color indicates higher spatial density) superimposed on the NPC architecture (blue). Spatial dimension of effective transport route is marked in nanometer. C, the cytoplasmic side of the NPC. N, the nucleoplasmic side of the NPC.
(MOV)
