# Supplementary figures and images for: Role of Molecular Charge in Nucleocytoplasmic Transport
Source: PLoS One. 2014 Feb 18;9(2):e88792. doi: 10.1371/journal.pone.0088792 (PMC3928296; doi:10.1371/journal.pone.0088792)

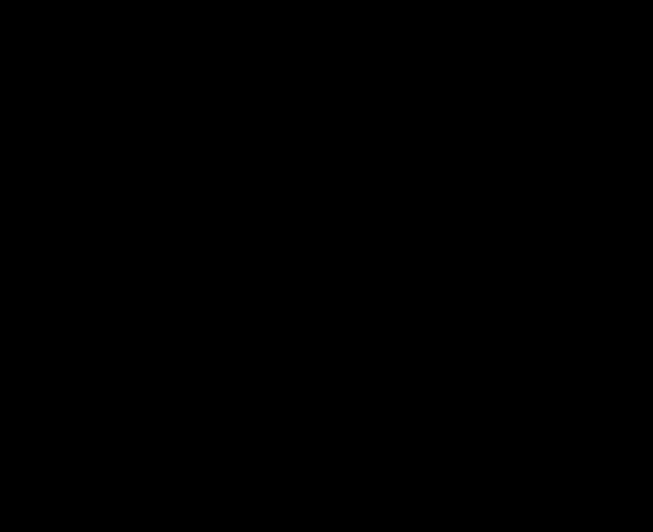

Supplement: Figure S1 — Determination of the hydrodynamic sizes of all GFP candidates by dynamic light scatting. 500 µL of 1 mg/mL of each candidate in PBS buffer was loaded onto Malvern Zetasizer Nano DLS for the measurements. The size distribution by intensity, mass and volume were measured multiple times and averaged. The average sizes were determined to be 5.9±0.7 nm, 6.1±1.1 nm and 5.8±0.9 nm for −30GFP (blue), −7GFP (green) and +36GFP (red). The number in the bracket is the diameter with s. d. in nanometer. (TIF) [file pone.0088792.s001.tif]

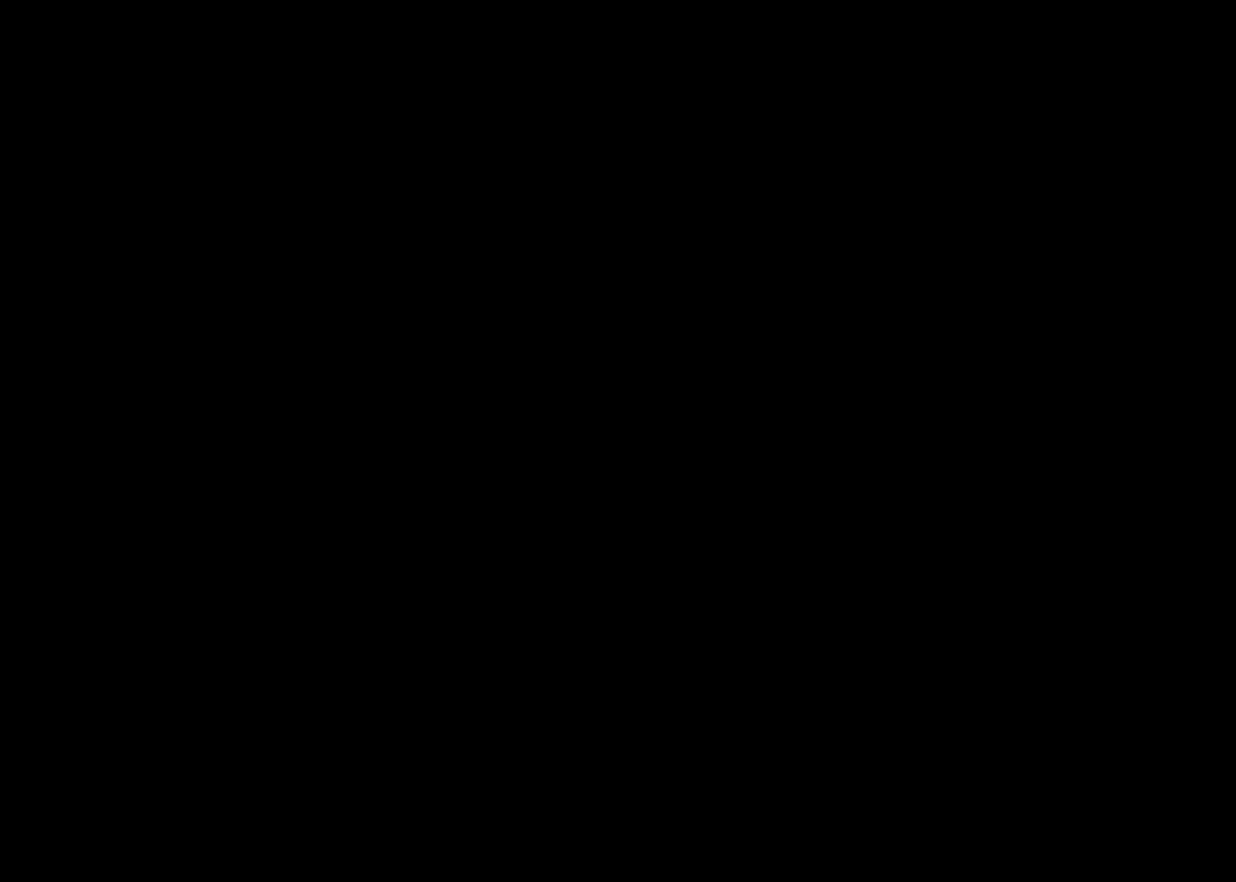

Supplement: Figure S2 — Typical single-molecule trajectories of nucleocytoplasmic transport events of signal-independent and signal-dependent GFPs obtained by SPEED microscopy. (A) A typical nuclear import event of supercharged −30GFP molecules from the cytoplasm to the nucleus. First, a single GFP-NPC (green spot) was visualized in the illumination volume. Then, a single fluorescent −30GFP molecule (red spot) entered the illumination volume, started in the cytoplasm (C), interacted with the NPC and entered the nucleus (N). Numbers denote time in millisecond. Scale bar: 1 µm. (B) Single-molecule trajectories of the import event in A. Based on the centroid (red dot) and the dimensions of the NPC, the spatial locations of Imp β1 molecule from 0.8 ms to 3.6 ms was within the NPC. (C and D) Individual video frames and the corresponding single-molecule trajectories of a typical export event for −30GFP. (E and F) Individual video frames and the corresponding single-molecule trajectories of a typical import event for the −30GFP import cargo complex. (TIF) [file pone.0088792.s002.tif]

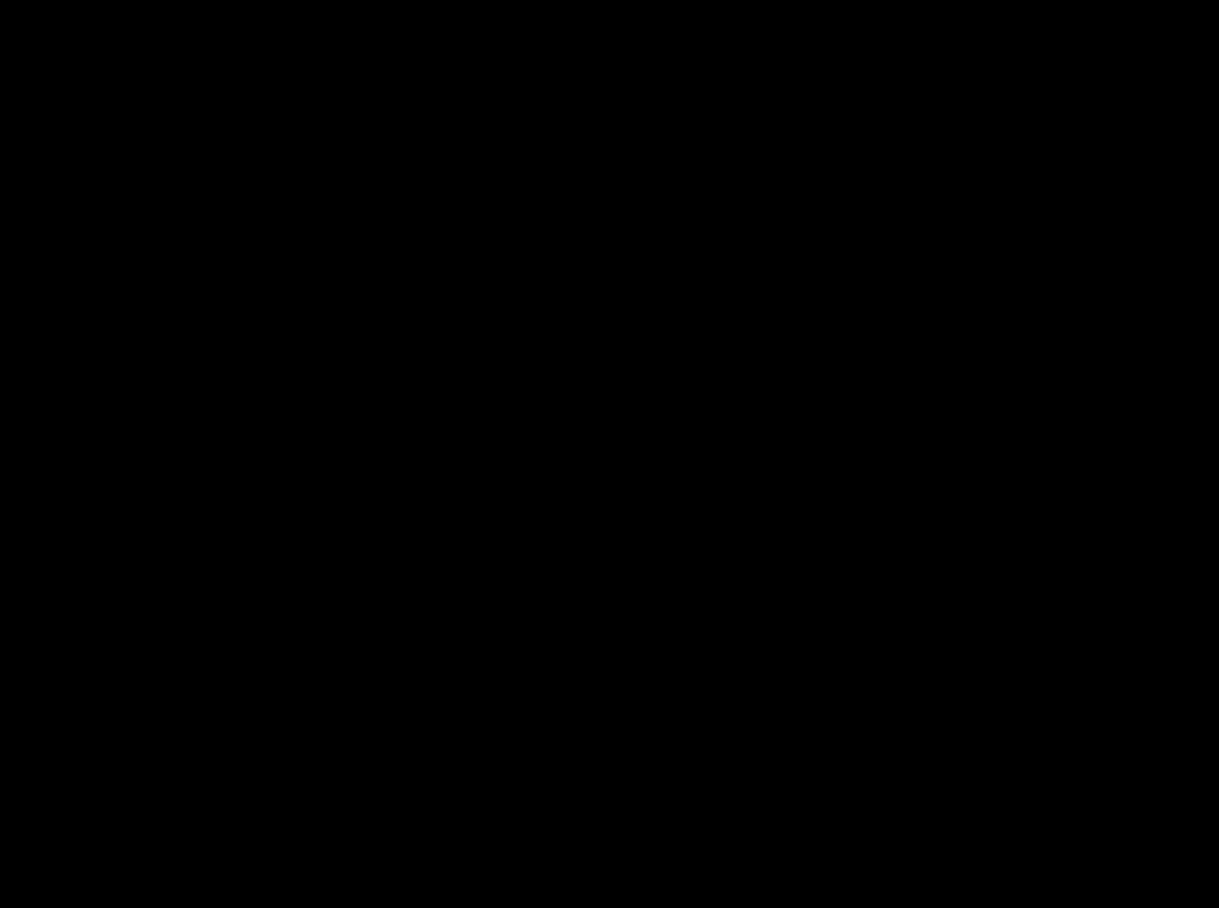

Supplement: Figure S3 — Typical single-molecule trajectories of nucleocytoplasmic transport events of rpL23 obtained by SPEED microscopy. (A and B) Individual video frames and single-molecule trajectories for typical import event for native ribosomal protein rpL23. (C and D) Individual frames and the corresponding single-molecule trajectories of a typical export even for rpL23. (E and F) Individual frames and trajectories for a typical import event for the import complex rpL23-Imp β1. (TIF) [file pone.0088792.s003.tif]

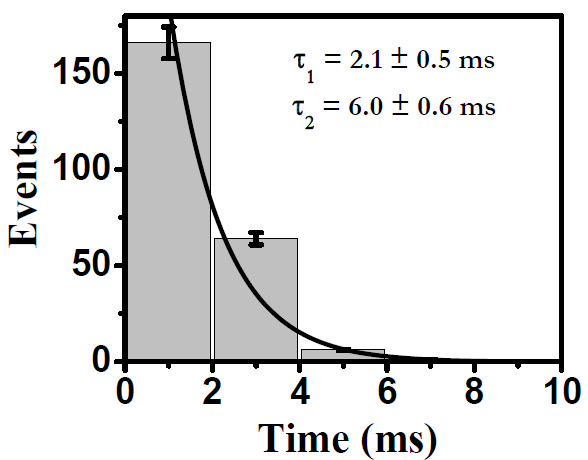

Supplement: Figure S4 — Nuclear import times of rpL23-mCherry in live HeLa cells. Histogram of 237 import events for rpL23-mCherry in live cells is fit well by a double exponential decay function generating two distinct import times of 2.1±0.5 ms and 6.0±0.6 ms. (TIF) [file pone.0088792.s004.tif]
